# The Potential Role of Thyroid Hormone Therapy in Neural Progenitor Cell Differentiation and Its Impact on Neurodevelopmental Disorders

**DOI:** 10.1007/s12035-023-03751-8

**Published:** 2023-11-22

**Authors:** Salam Salloum-Asfar, Kyung Chul Shin, Rowaida Z. Taha, Shahryar Khattak, Yongsoo Park, Sara A. Abdulla

**Affiliations:** 1 Neurological Disorders Research Center, Qatar Biomedical Research Institute, Hamad Bin Khalifa University, Qatar Foundation, Doha, Qatar; 2 BESE and KAUST Smart-Health Initiative (KSHI), King Abdullah University of Science and Technology (KAUST), Thuwal, Saudi Arabia

**Keywords:** Thyroid hormone, Neuronal differentiation, Neurodevelopmental disorders, Cortical differentiation, ADHD

## Abstract

Thyroid hormone (T3) plays a vital role in brain development and its dysregulation can impact behavior, nervous system function, and cognitive development. Large case-cohort studies have associated abnormal maternal T3 during early pregnancy to epilepsy, autism, and attention deficit hyperactivity disorder (ADHD) in children. Recent experimental findings have also shown T3’s influence on the fate of neural precursor cells and raise the question of its convergence with embryonic neural progenitors. Our objective was to investigate how T3 treatment affects neuronal development and functionality at the cellular level. In vitro experiments using neural precursor cells (NPCs) measured cell growth and numbers after exposure to varying T3 concentrations. Time points included week 0 (W0) representing NPCs treated with 100 nM T3 for 5 days, and differentiated cortical neurons assessed at weeks 3 (W3), 6 (W6), and 8 (W8). Techniques such as single-cell calcium imaging and whole-cell patch clamp were utilized to evaluate neuronal activity and function. IHC staining detected mature neuron markers, and RNA sequencing enabled molecular profiling. W6 and W8 neurons exhibited higher action potential frequencies, with W6 showing increased peak amplitudes and shortened inter-spike intervals by 50%, indicating enhanced activity. Transcriptomic analysis revealed that W6 T3-treated neurons formed a distinct cluster, suggesting accelerated maturation. Comparison with the whole transcriptome further unveiled a correlation between W6 neurons treated with T3 and neuronal regulatory elements associated with autism and ADHD. These findings provide insights into T3’s impact on neuronal development and potential mechanisms of T3 dysregulation and neurodevelopmental disorders.

**Supplementary Information:**

The online version contains supplementary material available at 10.1007/s12035-023-03751-8.

## Introduction

During the development of the brain, thyroid hormones (TH) play an essential role in neurogenesis, neuronal migration, neuronal differentiation, myelination, and synaptogenesis. THs bind to TH receptors (TRs), which influence gene expression positively or negatively. Triiodothyronine (T3) is the main biologically active form of THs. The development of a fetus critically depends on the presence of maternal TH during the first trimester of pregnancy. Later, the fetus is able to produce its own, but still relies on the mother to some extent [1]. There could be serious consequences for the child if the mother’s supply is inadequate and could have a detrimental effect on the child’s behavior, development, and cognitive abilities [2].

Hypothyroidism is one of the most common endocrine disorders that affects women more frequently and its incidence increases with age [3]. The findings of epidemiological studies suggest that maternal hypothyroidism during pregnancy changes the ratio of white to grey matter and is associated with a lower overall IQ and a lower level of verbal and motor performance in the child [4–8].

An abnormal thyroid function in early pregnancy was associated with epilepsy, autism spectrum disorders, and ADHD in children from the Danish National Birth Cohort case-cohort study, but the associations were different depending on the type of exposure and child age and gender [9]. Additionally, existing hypothyroidism treatment strategies do not provide optimal results. Many women under thyroid hormone therapy are either overtreated or undertreated because of drug interactions, malabsorption syndromes, pancreatic and liver disorders, autoimmune gastritis, high-fiber diets, or non-compliance with TH therapy. A variety of factors may affect serum thyroid-stimulating hormone (TSH) levels, including age, etiology of hypothyroidism, concomitant medications, and concomitant illnesses, which underscores the need for individualizing dosages.

Cognitive and sensorimotor functions are mediated by the human cerebral cortex, with TH deficiency during pregnancy or childhood widely recognized as a leading cause of intellectual disability. It is therefore important to ensure optimal thyroid function during pregnancy. There is a need for further research on the subtypes and severity of maternal thyroid function, as well as alternative outcomes of child development [10, 11]. It should be noted, however, that pregnant women suffering from thyroid disease do not always experience symptoms, and when they do, these symptoms may be attributed to the pregnancy itself. The importance of accurate laboratory assessment of maternal thyroid function increases in these situations [12, 13]. There is a need in the immediate future for a deeper understanding of the pathophysiology of psychiatric diseases, which will lead to the development of efficient and customized thyroid treatment plans.

Furthermore, recent research indicates that TH is an important regulator of neural stem cell fate determination, thereby raising the question of whether it also acts on similar pathways in embryonic neural progenitors [14]. Neural precursor cell (NPC) differentiation is modulated by a variety of internal and external signals, with THs playing an important role in promoting neuroblast development. To understand how adult neurogenesis is altered in physiological and pathological conditions, it is necessary to understand how homeostatic controls control neuronal and glial cell fate decisions. The objective of our study was to examine the effects of overtreated hypothyroidism state (high doses of T3 treatment) on neural functionality and the development of neural precursor cells (CRTD5-NPCs) to differentiated cortical neurons. Using an established in vitro system that mimics the development of neural precursor cells into functional cortical neuronal circuits, we differentiated NPCs into cortical excitatory neurons for 8 weeks. Using electrophysiological recordings and whole transcriptomic analysis, we found that W6 neurons treated with T3 show hyperactivity of neurons that might represent the phenotype of ASD and ADHD, suggesting that high-dose T3-treated neurons can be a model system to investigate the pathophysiology of ASD and ADHD.

## Results

### Activity of Voltage-Gated Calcium Channels in T3-Treated Human iPSC-Derived Cortical Neurons

T3 is critical for brain development and neuronal differentiation [15]. However, the molecular and cellular mechanisms of how dysregulation of T3 levels affect neuronal differentiation remain unclear. Using human iPSC-derived cortical neurons, we investigated the effect of a hypothyroidism state (high doses of T3) on human neuronal differentiation. As reported previously [16], we have generated human iPSC-derived cortical neurons and validated the functional maturation of these neurons differentiated for 8 weeks using the whole-cell patch-clamp technique and single-cell calcium imaging; i.e., human iPSC-derived cortical neurons after 6 and 8 weeks of differentiation become functionally active and mature, whereas 3-week-old cortical neurons are relatively immature.

Given that calcium ions (Ca^2+^) regulate gene expression and neuronal differentiation [17], we tested whether high doses of T3 lead to any changes in a Ca^2+^ level in human iPSC-derived cortical neurons (Fig. 3). Neurons were treated with a high dose of T3 during differentiation from NPCs to mature cortical neurons for up to 8 weeks. Intriguingly, a high dose of T3 slightly increased basal Ca^2+^ level in cortical neurons differentiated for 3 weeks, whereas basal Ca^2+^ level was decreased in 6-week-old cortical neurons (Fig. 3B–F); a high dose of T3 showed little effect on basal Ca^2+^ level in 8-week-old cortical neurons.

Next, we examined the effect of T3 on the activity of voltage-gated calcium channels (VGCCs) that mediate calcium influx to trigger vesicle fusion and neurotransmitter release in mature neurons. The activity of VGCCs represents neuronal differentiation and maturity of neurons, and we previously observed that calcium influx through VGCCs increases as human iPSC-derived cortical neurons become mature and electrically functional; L-type is one of the dominant VGCCs in human iPSC-derived cortical neurons [16]. A high dose of T3 did not significantly affect calcium influx stimulated by 50 mM KCl that depolarize the membrane potential and activate VGCCs in cortical neurons differentiated for 3, 6, and 8 weeks (Fig. 3G). The percentage of neurons responding to 50 mM KCl stimulation increased as cortical neurons matured over 8 weeks of differentiation, but a high dose of T3 had no effect on this percentage of neurons responding to KCl stimulation (Fig. 3H), suggesting that activity of VGCCs is not affected by high dose of T3 during neuronal differentiation.

### High Dose of T3 Treatment Causes Hyperactivity of Human iPSC-Derived Cortical Neurons

We further studied the functional activity of human iPSC-derived cortical neurons treated with a high dose of T3 by using the whole-cell patch-clamp technique (Fig. 4). Generation of action potential (AP) represents the neuronal activity of neurons, and we found that cortical neurons differentiated for 6 and 8 weeks generate multiple and repetitive action potentials; the percentage of neurons generating multiple APs increases as neurons differentiate, confirming the maturity of neurons [16].

Cortical neurons at week 3 mainly generate only a single AP, suggesting that these neurons are immature (Fig. 4A, B). Moreover, a high dose of T3 had no effect on the AP frequency and the peak amplitude of the AP in 3-week-old cortical neurons (Fig. 4A–C). However, the AP frequency was significantly increased by a high dose of T3 in 6-week-old human iPSC-derived cortical neurons (Fig. 4D, E). The peak amplitude of the AP was increased and the inter-spike interval (ISI), the time between subsequent APs, was decreased in high-dose T3-treated W6 cortical neurons (Fig. 4F, G). A high dose of T3 also resulted in the increase of the AP frequency in W8 cortical neurons (Fig. 4H, I), but the peak and ISI of the AP remained unaffected (Fig. 4J, K). The neuronal activity of W6 cortical neurons treated with a high dose of T3 is comparable with control W8 cortical neurons, suggesting that T3 might advance neuronal differentiation (Fig. 4E, I). W3 cortical neurons generate only a single AP; thus, there are no ISI data available. Taken together, our data provide evidence that a high dose of T3 enhances neuronal maturity and causes hyperactivity by increasing the AP frequency in mature cortical neurons.

### Increased Growth of Dendrites and Soma Cells with T3 Treatment as Neurons Differentiate Over Time

In all weeks, T3-exposed neurons exhibit increased growth of dendrites and soma cell bodies, abnormal morphology, and increased MAP2 staining on individual cells as well as within cells (Fig. 5A). There is some evidence that the increase in soma is correlated with the increase in the speed at which an action potential travels down an axon. There may be a non-uniform distribution of demand for mitochondrial functions such as ATP synthesis and calcium homeostasis due to the size and asymmetry of neurons. To maintain membrane excitability and to perform complex processes of neurotransmission and plasticity, neurons rely heavily on mitochondrial function. Although staining with MitoTracker red did not reveal significant differences in mitochondrial intensity (Fig. 5B), we are exploring the nuclear-to-cytoplasmic fluorescence ratio, as well as the three functionally distinct regions of the axon: the branch points, the distal axons, and the remainder of the axon shaft.

### Whole-Transcriptome Analysis with Total RNA Sequencing Detects Coding Plus Multiple Forms of Transcripts

Total RNA sequencing detected multiple forms of coding and non-coding RNA as neurons differentiate, regardless of whether T3 treatment is applied (Fig. 6A). The data was reprocessed by removing duplicates, adapters trimming some of the reads, mapping to the reference, and finally quantification. It is imperative that raw data be subjected to rigorous quality control (QC) procedures before being analyzed and downstream analyzed. Moreover, this is important to avoid the distortion of analytical results and erroneous conclusions. We controlled and predetermined the read depth before sequencing, resulting in a range of 93 to 147 million reads per sample (Fig. 6B). In order to minimize the probability of false results, we used a minimum of 93 M reads per sample in order to capture rare RNA sequences, detect low abundance variants, and detect alternative splicings. The reads from our sample were mapped to more than 95% of the human genome (Fig. 6B). As the neurons differentiate, the total gene counts oscillate between 95 and 85% corresponding to NPCs and W3 neurons, and 70% corresponding to W6 and W8 neurons (Fig. 6C). There is a qualitative difference between samples in the distribution of reads mapping to exonic, intronic, and intergenic regions (Fig. 6C). Consequently, the number of reads mapping to intergenic regions increased significantly at W6 and W8 as well as with the addition of T3, indicating that T3 may affect the splicing or non-coding RNAs expressed at this critical period of neuronal development. It is common for intergenic genes to be novel transcripts that have not yet been described in the Ensembl database. There are approximately 80% of transcripts from unannotated intergenic regions that are derived from the fuzzy transcription of existing genes; the remaining transcripts originate mostly from putative long non-coding RNA loci that are rarely spliced. A transcriptional start site, chromatin signature, and RNA polymerase II phosphorylation state may alter these characteristics. As we are sequencing the entire transcriptome, we should expect a wide variety of RNA biotype expression, with protein-coding genes exhibiting higher levels of expression than long non-coding RNAs (lncRNAs). There is also subcellular compartmental enrichment for other gene classes, such as pseudogenes and small annotated ncRNAs (Fig. 6D).

### Gene Expression Differences Between T3-Treated and Control Neurons in W6

Using multidimensional scaling (PCA) analysis, we examined the source of variation within the samples by unsupervised clustering of normalized gene expression data. There was no removal of outlier samples from the analysis. The NPCs and W3 neurons are clustered in two different groups and are primarily separated from the rest of the mature neurons at W6 and W8, which indicates that the maturation state accounts for the largest proportion of the overall variability. A significant difference exists between neurons at W6 and W8. W6 neurons that were treated with T3 formed an independent cluster, which is consistent with patch-clamp results showing a higher rate of maturation in W6 neurons with T3 treatment. W6-treated cells exhibit more changes than W3 or W8-treated cells. Our next step was to quantify differentially expressed genes (DEGs) in neurons treated with the CLC Genomics Workbench 22.0.2 statistical differential expression test based on a general linear model with a negative binomial distribution (http://digitalinsights.qiagen.com/) for 3 weeks (W3), 6 weeks (W6), and 8 weeks (W8) and their time-matched non-treated control groups. Based on an FDR of 5%, 997, 1918, and 34 DEG were differentially expressed at W3, W6, and W8 with T3 treatment, respectively (Fig. 3F and G). According to the Venn diagram illustrating the full range of possible intersections between different groups (Fig. 6G), 367 DEGs were upregulated and 530 downregulated in W3. A total of 821 DEGs were upregulated in W6, whereas only four DEGs were upregulated and ten downregulated in W8 (Fig. 6H).

### EIF2A Signaling Pathway in W6

Focusing on dysregulated DEGs of W6-T3-treated neurons, we used ingenuity pathway analysis, reference set “Ingenuity Knowledge Base (Genes Only),” and B-H multiple testing correction *p*-value to identify the enrichment pathways involved. Five biofunctions were strongly enriched for “molecular and cellular functions” related to (1) protein synthesis (*p*-value range 1.09E − 06–7.69E − 39; #Molecules: 85), (2) RNA damage and repair (*p*-value range 2.63E − 08–9.28E − 26; #Molecules: 36), (3) gene expression (*p*-value range 4.65E − 06–5.69E − 2; #Molecules: 95), (4) cell death and survival (*p*-value range 2.98E − 06–6.65E − 17; #Molecules: 120), and (5) RNA post-transcriptional modification (*p*-value range 1.22E − 06–3.17E − 14; #Molecules: 34). When focusing on the canonical pathways involved, the 3 top category canonical pathway are cellular growth, proliferation, and development; cellular stress and injury and intracellular and second messenger signaling were the most significant pathway name involved is EIF2 signaling (Fig. 5A and B). The complete list of differentially expressed genes and CLC gene ontology analysis results are provided in supplementary Tables 1 and 2. Moreover, the plot of the predicted activity of the EIF2 signaling pathway across 16,180 OmicSoft analyses identifies an inhibited and significant gene expression pattern on EIF2 signaling with a strong *z*-score of − 3.710. The position on the chart therefore describes the inhibited and significant predicted activity of the EIF2 signaling pathway in the analysis across 16,180 OmicSoft analyses (Fig. 5C). These results reinforce our analysis.

### Match Analysis of Transcriptomes Revealed a Correlation Between T3 Treatment and Neuronal Regulatory Elements in ASD and ADHD

The IPA Analysis Match automatically identified curated IPA datasets from public sources using QIAGEN OmicSoft Suite with significant similarities and differences to W6 neurons treated with T3 based on a shared pattern of predicted UR, CP, and DE. The 14 most similar and opposite analyses were selected, 12 of which came from “ADHD or ASD.” The analyses that matched it are enriched for analyses derived from neurons related to the 12 datasets of ADHD and ASD and that unmatched it are enriched to Alzheimer dataset (Fig. 5E). It was enriched for analyses derived from neurons associated with the 12 ADHD and ASD datasets with *z*-scores greater than ~ 30 and *p*-values between 1.03E − 12–6.66E − 32 (Table 1). There are, however, also matches against the analyses, related to the Alzheimer dataset with a *z*-score less than − 30 and a *p*-value between 3.39E − 05–1.24E − 38 (Table 1). Based on our findings, T3 treatment alters the transcriptome of iPSC-derived cortical neurons and plays an important role in the maturation and neurodevelopment of these cells. Interestingly, there was a correlation between this dataset and transcriptomic datasets related to ADHD and autism.

**Table 1:**
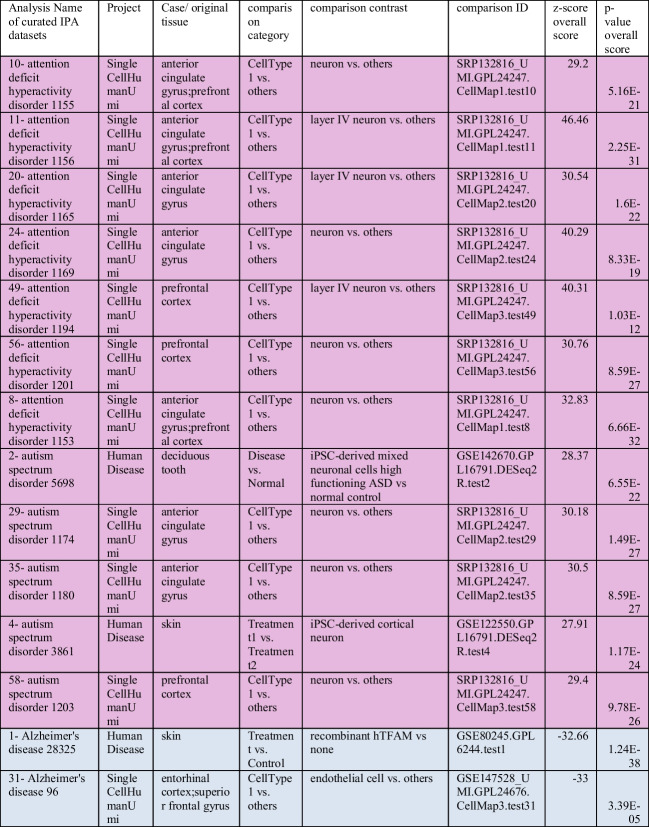
IPA Analysis Match containing curated IPA datasets (from public sources using QIAGEN OmicSoft Suite: SRA, GEO, Array Express, TCGA (by mutational status), LINCS, GTEx, ENCODE Consortium) with significant similarities and differences to W6 neurons treated with T3

## Discussion

The deficiency of T3 during fetal and postnatal development may result in retarded brain maturation, intellectual deficits, and in some cases neurological impairment. During development, thyroid hormone levels are deficient in the brain due to iodine deficiency, congenital hypothyroidism, as well as maternal hypothyroidism and hypothyroxinemia. Furthermore, elevated levels of thyroid-stimulating hormone (TSH) during pregnancy are associated with an increased risk of preterm delivery, placental abruption, fetal death, and impaired neurological development in the developing child. It is less clear whether and how TH regulates brain development during the initial stages of neural circuit formation. It was previously described that thyroid hormone stimulates neurogenesis, neural differentiation, and the formation of dendritic arbors in the tadpole visual system. The authors Thompson and Cline [18] have suggested that endogenous TH can be used to affect neurogenesis at developmental stages relevant to circuit assembly by influencing neural progenitor cell proliferation and differentiation, in addition to enhancing the formation of dendritic arbors on neurons.

This study supports the hypothesis that fetal brain development is negatively affected by untreated maternal thyroid dysfunction present during pregnancy. To gain a better understanding of ASD, it is necessary to identify the contributing factors. In addition to chromosomal abnormalities, deletions and duplications, single-gene disorders, and variants, several genetic factors and mechanisms contribute to the development of ASD. Moreover, the onset of ASD is associated with a wide range of environmental and metabolic abnormalities.

A novel aspect of this study is that it examines the NPCs stage and throughout cortical neuronal differentiation to mature neurons. High T3 treatment contributes to neuronal maturation. Due to the compartmentalized nature of neurons, regulating gene expression for appropriate cellular function poses a unique challenge for cells. The correlation between mRNA levels and soma size in single identified neurons provides evidence for compartment-specific regulation of gene expression [19]. Based on our findings, T3 treatment alters the transcriptome of iPSC-derived cortical neurons and plays an important role in the maturation and neurodevelopment of these cells. Moreover, we observed a correlation between this dataset and transcriptomic datasets related to ADHD and autism.

Furthermore, we will be expanding on our treatment approaches, and on different cell lines and exploring the three functionally distinct regions: axonal branch points, distal axons, and the remaining axon shaft.

There has to be a tight regulation, especially during the early weeks of embryonic development, as imbalances to TH are critical in prenatal life, with the fetus highly sensitive to its changes [20]. In the presence of an abnormal level of TH in the body, behavior, the functioning of the nervous system, and cognitive development may be adversely affected. Although most thyroid diseases are easily diagnosed and fully treatable, there are still ways to improve the thyroid health of patients. The promise of precision medicine is still much in the future. A personalized diagnostic and therapeutic approach that considers both a patient’s genomic profile and their environment should arouse significant anticipation. This new medical paradigm will be explored by clinicians who care for patients with thyroid dysfunction as the personalized medicine initiative unfolds. While there are challenges ahead, the potential for clinical benefit is undeniable, and momentum is building [21, 22].

In W6 T3-treated neurons, EIF2 signaling is the most significant pathway involved. eIF2α is responsible for the consolidation of memories through excitatory and somatostatin neurons [23]. Eukaryotic initiation factor 2 (eIF2) is arguably the most important initiation factor involved in neuron-specific translation regulation.

VGCCs regulate the generation and propagation of action potentials in excitable cells, including neurons and muscle cells [24]. The influx of calcium ions triggers various cellular processes, such as neurotransmitter release in neurons, muscle contraction in muscle cells, and gene expression in other cell types [24]. In neurons, the influx of calcium ions through VGCCs triggers the release of neurotransmitters into the synaptic cleft, transmitting the action potential and triggering downstream cellular events [24]. We observed that T3 has little effects on the calcium influx through VGCCs (Fig. 3G), despite the increase of action potential frequency in T3-treated neurons differentiated for 6 weeks (Fig. 4D–G). Our data suggest that the hyperactivity of T3-treated neurons may be due to alterations in other ion channels, such as Na^+^ and K^+^ ion channels. Various reports have previously indicated the effects of thyroid hormone on neuronal excitability related to changed properties of Na^+^ and K^+^ ion channels [25] that could lead to hyperactivity in neurons [26].

Another possibility is that T3 affects intracellular signaling pathways or gene expression patterns, which could have downstream effects on neuronal excitability [27, 28]. T3 regulates neuronal excitability, firing properties, and frequency of spontaneous excitatory postsynaptic currents (EPSCs) of neurons, providing evidence that T3 can affect neuronal excitability through alterations in intracellular signaling pathways[29].

T3 can also affect intracellular calcium levels by regulating the expression and activity of calcium-binding proteins [30]. We observed that T3 regulates basal calcium levels independently of VGCCs (Fig. 3F). T3 might change the intracellular calcium levels through the gene expression and activity of calcium-binding proteins [31].

Overall, our data propose that the hyperactivity of T3-treated neurons is likely due to alterations in multiple ion channels and intracellular signaling pathways, rather than solely due to changes in VGCC activity. Further studies will be needed to fully elucidate the underlying mechanisms and implications of these findings, which may have important implications for the treatment of neurological disorders.

## Materials and Methods

### iPSC-CRTD5 Neural Precursor Cell (NPC) Culture and Maintenance

Human-induced pluripotent stem cell (hiPSC) line generated by the Stem Cell Engineering Facility CRTD5. CRTD5 NPCs from human pluripotent stem cells were obtained as described before [32]. NPC media consisted of DMEM/F12 with Glutamax (Gibco), N2 supplement (1X, Gibco), B27 (without vitamin A), Laminin (1 ul/ml, Gibco), NEAA (1X, Gibco), heparin (2 ug/ml) and fibroblast growth factor (bFGF; 10 ng/ml, Sigma-Aldrich). NPCs were grown on Poly-L-Ornithine/Laminin-coated plates. Cells were cultured at 37 °C in a humidified atmosphere at 5% CO_2_. Briefly, bFGF, N2 supplement, B27 supplement, non-essential amino acids, Glutamax, Laminin, and heparin. bFGF is essential for cell proliferation. N2 is a neuronal media supplement containing insulin, transferrin, putrescine, selenite, and progesterone. N2 supplement functions in the growth and expansion of NPCs as well as support differentiation of NPCs to neurons. B27 is a neuronal cell media supplement that helps in the survival, growth, and maturation of various neuronal cell types. Glutamax is a substitute for glutamine and helps cells in efficient energy metabolism and growth in turn reducing the harmful effects of ammonia on the cells. Non-essential amino acids are provided for cell survival/health and not to put a burden on the cell’s internal machinery to produce their own amino acids. Laminin is an extracellular matrix glycoprotein abundantly expressed in developing neural tissues. It functions to induce and promote neurite outgrowth in vitro neurons. Heparin helps in the proliferation of neuronal stem cells.

### Differentiation of iPSC-CRTD5 NPCs into Mature Cortical Neurons

Human pluripotent stem cell–derived cortical neurons were obtained as described before [32]. Briefly, NPCs before passage 6 were dissociated using StemPro™ Accutase™ Cell Dissociation Reagent (Thermo Fisher) and plated on Poly-Ornithine/Laminin-coated plates. BrainPhys™ Neuronal Culture Medium (Stem Cell Technologies) was used to differentiate CRTD5-NPCs to cortical neurons following the Stemcell Technologies recommended protocol. BrainPhys™ Neuronal Culture Medium is used for compatibility with the generated NPCs. Briefly, the procedure involves seeding the cells onto Poly-Ornithine/Laminin-coated plates at a density of 2 × 10^4–3 × 10^4 cells/cm^2^ in 0.5 ml of NPC media. The NPCs were cultured in NPC media till reaching confluency. The next day, the media was changed to complete neuronal differentiation media (BrainPhys™ Neuronal Medium + NeuroCult™ SM1 Neuronal Supplement from kit). Refresh/change half of the media (complete neuronal differentiation) every second day for 8 weeks at 37 °C in a humidified atmosphere at 5% CO_2_. For RNA experiments, 6-well plates were used. For immunohistochemistry and mitotracker experiments, cells were cultured in µ-Slide 8 well-chambered slides suitable for immunofluorescence and high-end microscopy (80826, Ibidi). For single-cell calcium imaging and whole-cell patch-clamp electrophysiology, 4-well plates with a surface area of 1.9 cm^2^ were employed.

### Thyroid Hormone T3 Treatment

**Fig. 1:**
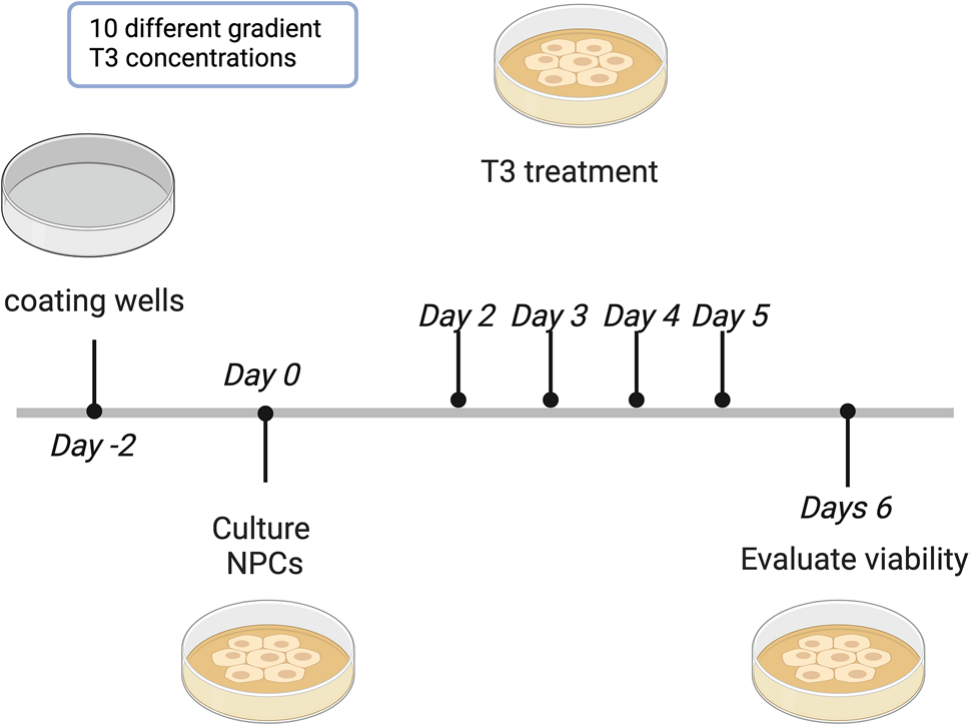
Time-course and gradient concentration of T3 on NPCs. This figure depicts the experimental design and results of the time-dependent assessment of T3 concentrations (ranging from 0 to 1000 nM)

**Fig. 2:**
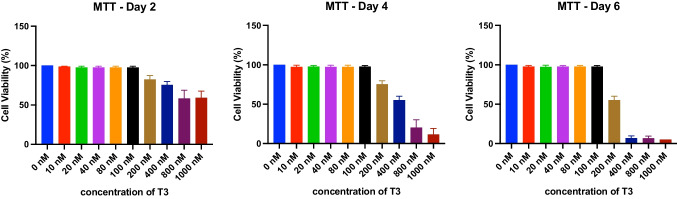
MTT CyQUANT™ Cell Proliferation Assay Kit results on days 2, 4, and 6. The graph illustrates the cell proliferation response to varying concentrations of T3 (ranging from 0 to 1000 nM) over the specified time points

L-3,3′,5-Triiodothyronine (T3), sodium salt (catalogue # 64245) was purchased from Sigma. A cytotoxicity evaluation was performed before and after exposure to 10 different T3 gradient concentrations (10–1000 nM) for a week (Fig. 1) using cytotoxicity assay using the MTT CyQUANT™ Cell Proliferation Assay Kit, Catalog #V13154. Moreover, to examine changes in the morphology of T3-treated CRTD5 NPCs and untreated control cells, inverted microscopy was performed. Based on the MTT values and results of inverted and fluorescence microscopy, thyroid hormone reduced cell viability in CRTD5 NPCs. One hundred nanometers of T3 was selected as the maximum concentration that cells can tolerate (Fig. 2).

### The Time-Course Study

**Fig. 3:**
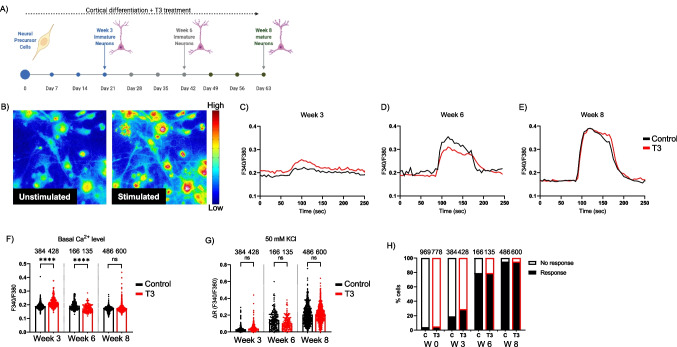
Single-cell calcium imaging to monitor neuronal activity in T3-treated hiPSC-derived neurons. **A** Time-course experiment overview. **B** Representative image of Fura-2-loaded hiPSC-derived neurons after 8 weeks of differentiation: left, before stimulation; right, 50 mM KCl stimulation. **C–E** Representative traces of intracellular calcium ions (Fura-2 F340/F380 ratio) in control (black) and high dose of T3-treated (red) neurons stimulated by 50 mM KCl for 2 min; 3 (**C**), 6 (**D**), and 8 (**E**) weeks of differentiation. **F** The intracellular basal Ca^2+^ levels in control (black) and T3 (red)-treated neurons differentiated for 3, 6, and 8 weeks. **G** Net changes of Ca^2+^ increase by 50 mM KCl. Data in **F**, **G** are means ± SEM, and the number of cells tested is shown from 3 ~ 4 independent differentiation. Kruskal–Wallis one-way ANOVA and Dunn’s post hoc test were used; ****p < 0.0001. **H** Percentage of neurons that evoke calcium influx in response to 50 mM KCl

**Fig. 4:**
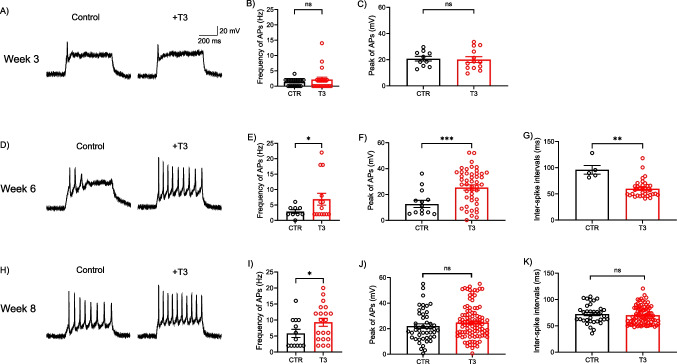
Electrophysiological activity of T3-treated hiPSC-derived neurons. Electrophysiological whole-cell patch-clamp recording from neurons differentiated for 3 (**A–C**), 6 (**D–G**), and 8 (**H–K**) weeks. **A**, **D**, **H** Representative traces of action potentials (AP) generated by injection of current pulses in a current-clamp mode. **B**, **E**, **I** AP frequency (Hz), the number of spikes per second. **C**, **F**, **J** AP peak (mV), the peak amplitude of AP. **G**, **K** Inter-spike interval (ISI), the time between subsequent APs. Data are means ± SEM from 3 ~ 4 independent differentiation. Unpaired one-tailed *t*-test with Welch’s correction; *p < 0.05; **p < 0.01

**Fig. 5:**
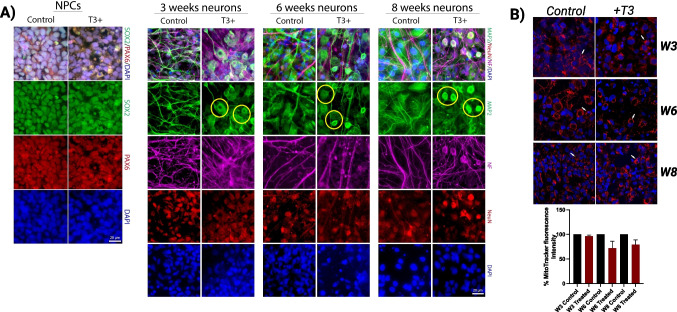
Expression of precursor and neuronal markers, and mitochondrial function of T3-treated hiPSC-derived neurons. **A** Immunostaining at NPCs stage shows the expression of SOX-2 (green) and PAX-6 (red) and hiPSC-derived neurons after 3, 6, and 8 weeks of differentiation week stages (Map-2 (green), neurofilament-H NF-H (pink), NeuN (red). Yellow circles correspond to abnormal morphology, and increased MAP2 staining on individual cells as well as within cells. **B** No differences in mitotracker fluorescence intensity and size using confocal microscopy. White arrows indicate the mitochondrial similar size (scale bars: 10 μm in all images). The blue channel corresponds to the Hoechst-stained cell nuclei (DAPI). In all panels scale bar = 10 µm. Laser scanning confocal microscopy using a TCS SP Leica microscope (Lasertechnik, GmbH)

**Fig. 6:**
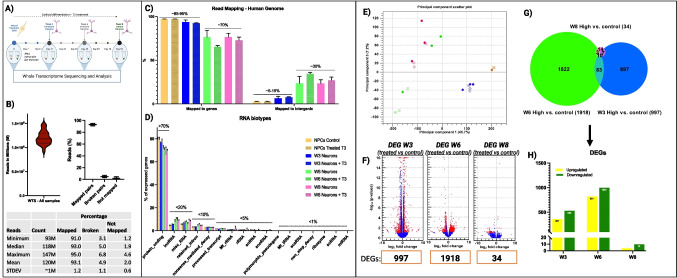
Whole-transcriptome analysis and differential expression analysis (DEG). **A** Multiple forms of coding and non-coding RNA as neurons differentiate, regardless of whether T3 treatment is applied. **B** The read depth before sequencing, resulting in a range of 93 to 147 million reads per sample. **C** Total gene count percentages corresponding to NPCs and W3, W6, and W8 neurons. **D** RNA biotype expression. **E** multidimensional scaling (PCA) analysis. **F** Volcano plots. **G** Venn diagram showing the total number of differentially expressed genes 3 weeks (W3), 6 weeks (W6), and 8 weeks (W8) vs. their time-matched non-treated control groups. **H** Upregulated and downregulated DEGs according to the Venn diagram

**Fig. 7:**
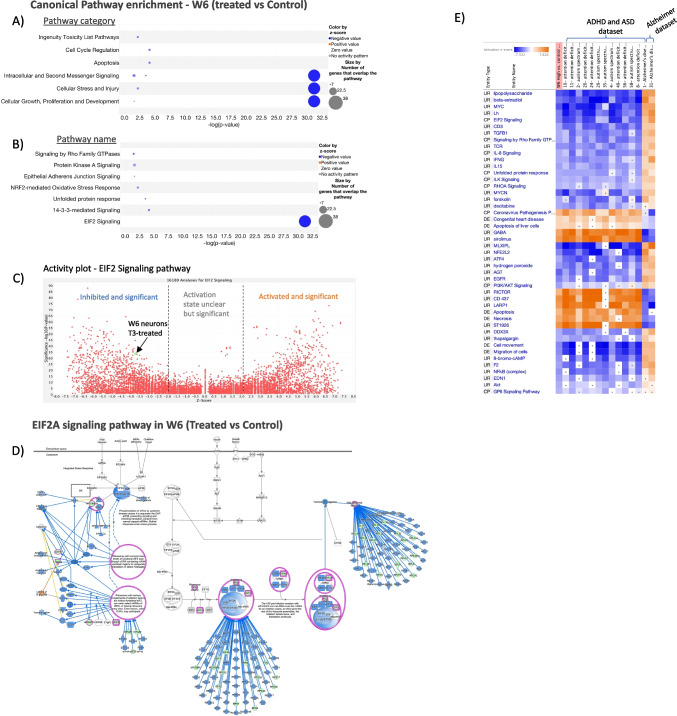
Pathway analysis of W6 T3-treated hiPSC-derived neurons. **A** Canonical pathway enrichment based on pathway category. **B** Canonical pathway enrichment based on pathway name. **C** Activity plot—EIF2 signaling pathway. **D** Identified EIF2A signaling pathway in W6 (treated vs control). Genes highlighted in blue have been tested B-H multiple testing correction *p*-value. **E** Analysis Match of hiPSC-derived neurons after 6 weeks of differentiation (treated vs control) against 100,000 + curated publicly available datasets in IPA

NPCs cultured in BrainPhys™ Neuronal Culture Medium to differentiate CRTD5-NPCs to cortical neurons were the starting point (named week 0). The time-course study consisted of 4 points of analysis: week 0 (W0), which represented NPCs after 5 days of T3 treatment, and differentiated cortical neurons measured at weeks 3 (W3), 6 (W6), and 8 (W8). Each time frame was performed in triplicates and included respective controls (Figs. 3, 4, 5, 6, and 7).

### Single-Cell Calcium Imaging

Single-cell calcium imaging experiments were performed as described previously [16]. Briefly, neurons were loaded with 3 µM of Fura-2 AM for 30 min and washed in NT for 30 min at room temperature. Dual excitation and emission were at 340/380 and 510 nm, respectively. Data acquisition was accomplished using EasyRatioPro software. KCl measuring 50 mM was applied to depolarize the membrane potential to evoke calcium influx through voltage-gated calcium channels.

### Whole-Cell Patch-Clamp Electrophysiology

Recordings of action potentials (Aps) using the conventional whole-cell configuration of the patch-clamp technique were carried out as described earlier [16]. Data acquisition, voltage control, and analysis were accomplished using software (HEKA Patchmaster). Neurons were perfused in the chamber with the normal Tyrode’s bath solution (mM): 143 NaCl, 5.4 KCl, 0.33 NaH2PO4, 0.5 MgCl2, 5 HEPES, 2 CaCl2, and 11 glucose; pH 7.4 adjusted with NaOH. We used patch pipettes with a resistance of 2–4 MΩ when filled with the internal solution (mM): 130 K-gluconate, 3 KCl, 2 MgCl2, 10 HEPES, 5 Na2ATP, 0.5 Na2GTP, 0.2 EGTA; pH 7.3 adjusted with KOH. For the recording of action potentials, a current-clamp mode was used under the conventional whole-cell configuration with a series of current steps from − 20 to + 60 pA for 500 ms. Signals were low-pass filtered with a cutoff frequency of 5 kHz and sampled at 10 kHz.

### Immunocytochemistry

Poly-L-Ornithine/Laminin cells grown on µ-Slide 8 well-chambered slide for cell culture (80826, Ibidi) were fixed for 15 min in 4% Paraformladehyde Solution in PBS (30525-89-4, ChemCruz-Fisher) and then washed three times with PBS-T (0.3% Triton X-100 in 1X PBS) by Sigma-Aldrich). The permeabilization and blocking steps were performed using 0.3% Triton X-100 (Sigma-Aldrich), 5% Donkey serum, and 2% of BSA (15260037, Thermo Fisher Scientific) in PBS for 1 h. In the blocking solution, primary antibodies were applied overnight at 4 °C. The samples were then washed with PBS-T three times (10 min each time), and the secondary antibodies and DAPI (10236276001, Sigma-Aldrich) were applied for 1 h at room temperature in 1X PBS. The samples were washed three times with 1X PBS for 10 min each. Prolong Gold Antifade (P36934, Thermo Fisher Scientific) was used to mount the cells. This study used the following primary antibodies: mouse anti-NESTIN (MAB-5326, EMD), rabbit anti-PAX6 (901301, Biolegend), goat anti-SOX2 (365823, Santa Cruz), anti-NF-H (801601, BioLegend), anti-MAP2 (ab92434, Abcam), and anti-NEUN (ab177487, Abcam). A secondary antibody was purchased from Thermo Fisher Scientific and was conjugated to AlexaFluor fluorochromes (1:1000). Zeiss Axio Observer Z1 inverted fluorescence microscope was used for fluorescence imaging. Briefly, their respective roles are as follows: SOX2 is a neural stem cell marker and is shown to be essential for neural stem cell self-renewal and multipotent differentiation. PAX6 is a transcription factor and is considered a neural stem cell and differentiation marker. The role of Pax6 is to provide signalling cues for the formation of the central nervous system. NF (neurofilament) is an axonal marker for neurons. The protein is responsible for the maintenance and structure of the neuronal cytoskeleton primarily in axons. MAP2 (microtubule-associated protein 2) is the dendritic marker of axons. The function is to maintain the neuronal cytoskeleton primarily in dendrites. NeuN (neuronal nuclear protein) is the protein encoded by the Fox3 gene. It is a widely used postmitotic neuronal marker for central nervous system neurons. Functionally, it is responsible for RNA splicing in neuronal nuclei.

### Mitotracker and Confocal Microscopy

Cells were cultured in µ-Slide 8 well-chambered slides for immunofluorescence and high-end microscopy (80826, Ibidi). According to the manufacturer’s instructions, MitoTrackerTM Red CMXRos (M7512, Thermo Fisher) was prepared as directed. The CMXRos dye was dissolved in DMSO at a concentration of 1 mM and stored at − 20 °C until it was needed. The working solution (250 nM) for staining differentiation cultures was prepared directly from the stock solution in complete differentiation media. The cells were incubated in the dark for 30 min at 37 °C in a 5% CO_2_ atmosphere. Confocal microscopy was used to examine the fluorescence mitochondria of living cells using a TCS SP Leica microscope (Lasertechnik, GmbH).

### RNA Extraction and Assessment of Purity, Integrity, and Quantity

Total RNA was extracted from 21 samples of CRTD5-derived NPCs and cortical mature and immature neurons using the Direct-zol RNA Micro Prep Extraction Kit according to the manufacturer’s instructions (2062, Zymo Research). RNA quality control was done by Bioanalyzer using RNA Nano 6000 assay and RNA quantity was quantified by Qubit 2.0 (Thermo Fisher Scientific, USA) to measure the RNA concentration. RNA purity was assessed by determination of the ratio for absorbance at 260 nm vs. absorbance at 280 nm (A260 nm/A280 nm) using a NanoDrop spectrophotometer and Agilent RNA 6000 Nano Kit (5067–1511, Agilent, CA, USA) as per the manufacturer’s instructions. The yields of RNA were measured using NanoDrop and Qubit. In addition, the integrity of the RNA was assessed using the 2100 Bioanalyzer by electrophoresis using the RIN-values obtained by gel electrophoresis.

### Whole-Transcriptome Library Preparation and Sequencing

The library was prepared using SMARTer® Stranded Total RNA-Seq Kit v2—Pico Input Mammalian (Cat #634414) following the manufacturer’s protocol. The quality control of libraries was performed using the Agilent 2100 bioanalyzer and the DNA high-sensitivity kit. The libraries were pooled into 5 pools, clustered on a cBot platform, and sequenced on an Illumina HiSeq 4000 at a minimum of 50 million paired-end reads (1 × 75 bp + 1 × 75 bp) per sample.

### Whole Transcriptome and Functional Annotation Analyses

RNA sequencing (RNA-seq) analysis was performed using CLC genomic workbench software and the downstream pathway analysis using ingenuity pathway analysis (IPA).

A pair of demultiplexed fastq files was generated and an initial quality control was conducted using FastQC. Using CLC Genomics Workbench 22 (Qiagen), paired fastq files were uploaded and aligned against the hg38 human reference genome. As a measure of transcript abundance, TPM (transcripts per million) mapped reads were used. In the following steps, the abundance data were subjected to differential gene expression to identify differentially expressed transcript genes (DEGs) using the built-in statistical analyses recommended in the CLC Genomics protocol with an absolute fold change (FC) of 1.5 and a FDR *p*-value cutoff of 0.05.

### Enriched Pathway Analysis Using IPA

Ingenuity Pathway Analysis IPA (Ingenuity Systems; www.ingenuity.com/) was used to identify DEGs (differentially expressed genes) at the biologically functional level. Functional annotations and regulatory network analysis were performed using upstream regulator analysis (URA), downstream effects analysis (DEA), mechanistic networks (MN), and causal network analysis (CNA) prediction algorithms. By utilizing a precise algorithm, IPA is able to predict functional regulatory networks from gene expression data and assign a significance score to each network according to its fit to the set of focus genes in the database. An indication of the likelihood that the network’s focal genes were discovered by chance is the *p*-value, which represents the negative log of *P*.

### IPA Analysis Match

As part of Analysis Match, more than 100,000 highly curated and quality-controlled disease and oncology datasets were reprocessed in IPA from sources such as SRA, GEO, Array Express, TCGA (by mutational status), LINCS, GTEx, ENCODE Consortium, and others. The datasets were acquired by QIAGEN from OmicSoft and are the “comparisons” found in DiseaseLand, OncoLand, SingleCellLand, and Normal Cells and Tissues, which represent various contrasts such as disease and normal, treatment versus non-treatment, and others. As a result, the raw *z*-score is calculated as a percentage of maximum, i.e., a signature that matches strongly has a raw *z*-score of 80%, whereas a signature that matches weakly may have a raw *z*-score of 20%. There are opposite-matched datasets when the *z*-score percentage is negative.

### Statistical Analysis

Data analysis was performed using OriginPro 2019 software (OriginLab Corporation, Northampton, MA, USA) and GraphPad Prism 9 (GraphPad Software, San Diego, CA, USA). Data are means ± standard error of mean (SEM). Kruskal–Wallis one-way ANOVA and Dunn’s post hoc test were used to determine any statistically significant differences between three or more independent groups. An unpaired one-tailed *t*-test with Welch’s correction was applied to estimate the statistical significance between the two groups. Probabilities of *p* < 0.05 were considered significant.

### Supplementary Information

ESM 1 (XLSX 6.29 mb)

ESM 2 (XLSX 17.8 kb)

## Data Availability

The datasets generated during and/or analyzed during the current study are available from the corresponding author on reasonable request.

## References

[1] Stricker R (2007). Evaluation of maternal thyroid function during pregnancy: the importance of using gestational age-specific reference intervals. Eur J Endocrinol.

[2] LaFranchi SH, Haddow JE, Hollowell JG (2005). Is thyroid inadequacy during gestation a risk factor for adverse pregnancy and developmental outcomes?. Thyroid.

[3] Yavuz DG (2017). Out-of-reference range thyroid-stimulating hormone levels in levothyroxine-treated primary hypothyroid patients: a multicenter observational study. Front Endocrinol.

[4] Casey BM (2005). Subclinical hypothyroidism and pregnancy outcomes. Obstet Gynecol.

[5] Allan W (2000). Maternal thyroid deficiency and pregnancy complications: implications for population screening. J Med Screen.

[6] Haddow JE (1999). Maternal thyroid deficiency during pregnancy and subsequent neuropsychological development of the child. N Engl J Med.

[7] Moog NK (2017). Influence of maternal thyroid hormones during gestation on fetal brain development. Neuroscience.

[8] Korevaar TI (2016). Association of maternal thyroid function during early pregnancy with offspring IQ and brain morphology in childhood: a population-based prospective cohort study. Lancet Diabetes Endocrinol.

[9] Andersen SL (2018). Maternal thyroid function in early pregnancy and child neurodevelopmental disorders: a Danish nationwide case-cohort study. Thyroid.

[10] Krieger TG (2019). Mutations in thyroid hormone receptor α1 cause premature neurogenesis and progenitor cell depletion in human cortical development. Proc Natl Acad Sci.

[11] Bath SC (2013). Effect of inadequate iodine status in UK pregnant women on cognitive outcomes in their children: results from the Avon Longitudinal Study of Parents and Children (ALSPAC). Lancet.

[12] LeBeau SO, Mandel SJ (2006). Thyroid disorders during pregnancy. Endocrinol Metab Clin.

[13] Brent GA (1997). Maternal thyroid function: interpretation of thyroid function tests in pregnancy. Clin Obstet Gynecol.

[14] Gothié J (2017). Adult neural stem cell fate is determined by thyroid hormone activation of mitochondrial metabolism. Mol Metab.

[15] Chen C (2012). Thyroid hormone promotes neuronal differentiation of embryonic neural stem cells by inhibiting STAT3 signaling through TRalpha1. Stem Cells Dev.

[16] Shin KC et al (2023) Deletion of TRPC6, an autism risk gene, induces hyperexcitability in cortical neurons derived from human pluripotent stem cells. Mol Neurobiol. 10.1007/s12035-023-03527-0 10.1007/s12035-023-03527-0 PMC10657791 37552395

[17] Toth AB, Shum AK, Prakriya M (2016). Regulation of neurogenesis by calcium signaling. Cell Calcium.

[18] Thompson CK, Cline HT (2016). Thyroid hormone acts locally to increase neurogenesis, neuronal differentiation, and dendritic arbor elaboration in the tadpole visual system. J Neurosci.

[19] Ransdell JL, Faust TB, Schulz DJ (2010). Correlated levels of mRNA and soma size in single identified neurons: evidence for compartment-specific regulation of gene expression. Front Mol Neurosci.

[20] Andersen SL (2014). Psychiatric disease in late adolescence and young adulthood. Foetal programming by maternal hypothyroidism?. Clin Endocrinol.

[21] Ettleson MD, Bianco AC (2020). Individualized therapy for hypothyroidism: is T4 enough for everyone?. J Clin Endocrinol Metab.

[22] Wolff TM, Dietrich JW, Müller MA (2022) Optimal hormone replacement therapy in hypothyroidism - a model predictive control approach. Front Endocrinol (Lausanne) 13:884018. 10.3389/fendo.2022.884018 10.3389/fendo.2022.884018 PMC9263720 35813623

[23] Sharma V (2020). eIF2α controls memory consolidation via excitatory and somatostatin neurons. Nature.

[24] Catterall WA (2011). Voltage-gated calcium channels. Cold Spring Harb Perspect Biol.

[25] Harrison AP, Clausen T (1998). Thyroid hormone-induced upregulation of Na+ channels and Na(+)-K+ pumps: implications for contractility. Am J Physiol.

[26] Hoffmann G, Dietzel ID (2004). Thyroid hormone regulates excitability in central neurons from postnatal rats. Neuroscience.

[27] Flavell SW, Greenberg ME (2008). Signaling mechanisms linking neuronal activity to gene expression and plasticity of the nervous system. Annu Rev Neurosci.

[28] Cheng SY, Leonard JL, Davis PJ (2010). Molecular aspects of thyroid hormone actions. Endocr Rev.

[29] Caria MA (2009). Thyroid hormone action: nongenomic modulation of neuronal excitability in the hippocampus. J Neuroendocrinol.

[30] Carr AN, Kranias EG (2002). Thyroid hormone regulation of calcium cycling proteins. Thyroid.

[31] Novak P, Soukup T (2011). Calsequestrin distribution, structure and function, its role in normal and pathological situations and the effect of thyroid hormones. Physiol Res.

[32] Khattak S (2015). Human induced pluripotent stem cell derived neurons as a model for Williams-Beuren syndrome. Mol Brain.

